# Misplacement of a Naso-Intestinal Feeding Tube Into the Pleural Space in a Patient With Nasopharyngeal Carcinoma: A Case Report

**DOI:** 10.7759/cureus.110142

**Published:** 2026-06-02

**Authors:** Mengliu Jiang, Pengkai Duan, Jiao Shen, Shao Yu, Xiao Guizhen

**Affiliations:** 1 Clinical Nutrition, General Hospital of Southern Theatre Command of PLA, Guangzhou, CHN; 2 Critical Care Medicine, General Hospital of Southern Theatre Command of PLA, Guangzhou, CHN; 3 General Medicine, General Hospital of Southern Theatre Command of PLA, Guangzhou, CHN; 4 Clinical Nutrition, Southern University of Science and Technology Hospital, Shenzhen, CHN

**Keywords:** enteral nutrition, intensive care unit, naso-intestinal feeding tube, nasopharyngeal carcinoma, pleural cavity, radiotherapy, tube misplacement

## Abstract

Postpyloric enteral feeding is recommended for ICU patients at high risk of aspiration. However, blind placement carries a rare yet potentially life-threatening risk of tube misplacement into the pleural cavity. We present a case in which blind insertion of a fine-bore naso-intestinal tube led to intrapleural malposition in an elderly patient with a history of radiotherapy for nasopharyngeal carcinoma (NPC). A 78-year-old Chinese man with severe dysphagia following radiotherapy for NPC underwent tracheotomy due to respiratory failure and was subsequently transferred to the ICU. The naso-intestinal tube was inserted blindly, and its position was considered appropriate based on limited fluid aspiration and auscultation. Nine hours later, chest radiography revealed that the tube had traversed through the trachea, entered the right lower-lobe bronchus, extended into the pleural cavity, and terminated mid-thorax. The tube was promptly removed and accurately repositioned into the stomach under laryngoscopic guidance. Blind postpyloric tube placement in patients with radiation-induced anatomical and neurological changes following NPC may result in silent intrapleural misplacement. Immediate pH testing combined with radiographic confirmation is crucial to prevent this potentially fatal complication.

## Introduction

Many ICU patients, unable to meet their nutritional needs orally, require feeding tube placement, including those undergoing endotracheal intubation or tracheostomy [1]. Patients at high risk of aspiration may benefit from early postpyloric feeding, as clinical guidelines strongly recommend for such critically ill populations [2,3]. Although bedside blind insertion of feeding tubes is simple, convenient, and widely used in routine clinical practice, this empirical approach still carries procedural risks [4]. A rare yet fatal adverse event is inadvertent pleural misplacement of the feeding tube, often associated with nonstandard placement techniques or disease-related anatomical abnormalities [5]. 

Nasopharyngeal carcinoma (NPC) is endemic in East and Southeast Asia, with radiotherapy as the primary curative treatment [6]. Radiotherapy induces soft tissue fibrosis, cervical muscle atrophy, and cranial nerve injury in NPC patients, impairing swallowing and blunting airway reflexes. These irreversible changes increase naso-intestinal tube placement risk, predisposing patients to silent tracheobronchial or pleural misplacement without obvious warning signs. Traditional bedside confirmation methods like gastric aspiration and auscultation lack sufficient accuracy to rule out intrathoracic misplacement. Literature on this complication in postradiotherapy patients remains scarce. This report describes a rare case, analyzes contributing factors and procedural pitfalls, and aims to provide guidance for safe tube placement in high-risk populations.

## Case presentation

A 78-year-old Chinese man was admitted to the hospital with a five-day history of fever accompanied by cough, sputum production, dysphagia, and choking. His medical history includes radiotherapy for nasopharyngeal cancer 12 years ago, as well as a two-year history of Alzheimer's disease and cerebral infarction. The patient is 165 cm tall and weighs 58 kg (BMI: 21.3 kg/m^2^). The patient had a history of radiotherapy for over six years for NPC and subsequently developed related complications, including cervical muscle atrophy and stiffness, upper airway stenosis, and impaired autonomous coughing ability.

The patient experienced a persistent fever. About a week after hospitalization, he developed aspiration, which was accompanied by increased sputum production and cognitive impairment. The patient exhibited unstable peripheral oxygen saturation (SpO_2_) levels. The SpO₂ transiently dropped to 80% and fluctuated between 80% and 86%, with no obvious improvement after sputum suction and increasing inspired oxygen concentration. He was unable to undergo tracheal intubation due to upper airway stenosis. Subsequently, the patient was transferred to the ICU for further management. A tracheotomy was performed three hours later, with butorphanol tartrate administered for analgesia. Eight hours after the tracheotomy, a naso-intestinal tube (FLOCARE BENGMARK®, CH10-145) was inserted "blindly" by the nurse. Prior to insertion, the required length of the tube was measured, and two marks were placed at 55 cm and 110 cm. The patient was placed in a semi-recumbent position, and the tube was then advanced smoothly to the first mark without resistance. The nurse attempted to confirm the tube's position by aspirating gastric fluid; however, only a small amount of fluid was obtained, and the pH value was not measured.

**Figure 1:**
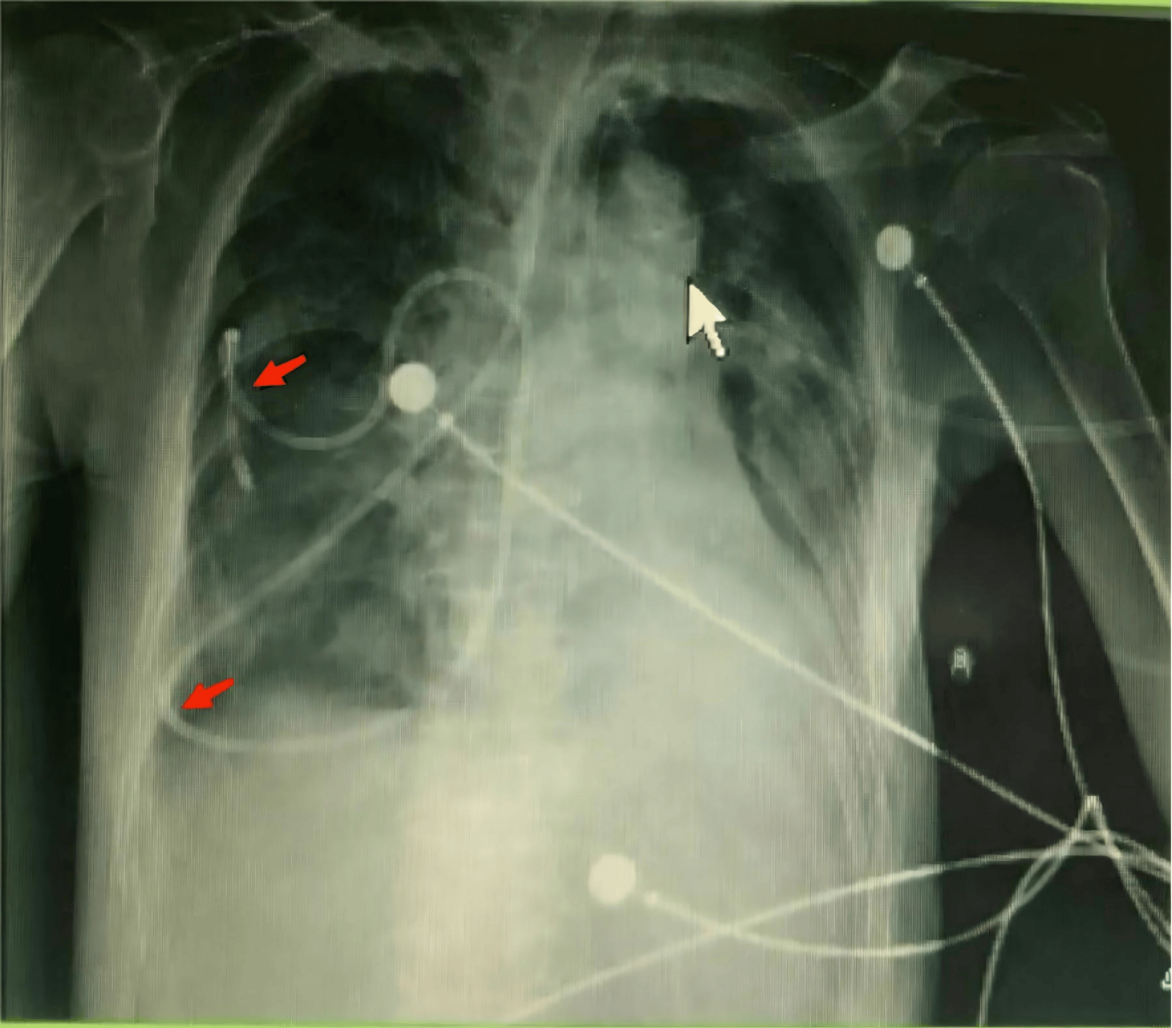
Bedside anteroposterior (AP) chest radiograph showing the naso-intestinal tube (red arrow) coiled in the right pleural cavity

To further verify the tube's position, auscultation was performed: an air bolus was injected into the tube while simultaneously listening with a stethoscope over the upper abdomen for an expulsion of air. A faint "gurgling" sound could be heard, leading to the mistaken assumption that the tube was positioned in the stomach. The tube was advanced to the 110 cm mark and securely fixed. The procedure proceeded smoothly without any obstruction, and the patient remained comfortable with no reported discomfort. Additionally, there was no decrease in SpO_2_. In accordance with the instructions for use, after placement of the tube in the stomach, the Bengmark spiral is expected to pass through the pylorus spontaneously within 8-12 hours owing to gastric motility. Nutrient solution is infused only after X-ray confirmation that the tube has reached the duodenum or jejunum at the preset depth. The findings revealed that the naso-intestinal tube passed through the trachea and bronchi, extended to the right lower lobe bronchus, entered the thoracic cavity, turned back at the right costophrenic angle, followed the pleural cavity with several folds, and ultimately terminated in the mid-thoracic region. Additionally, a patchy area of increased density was observed in the left lower lung field (Figure 1).

**Figure 2:**
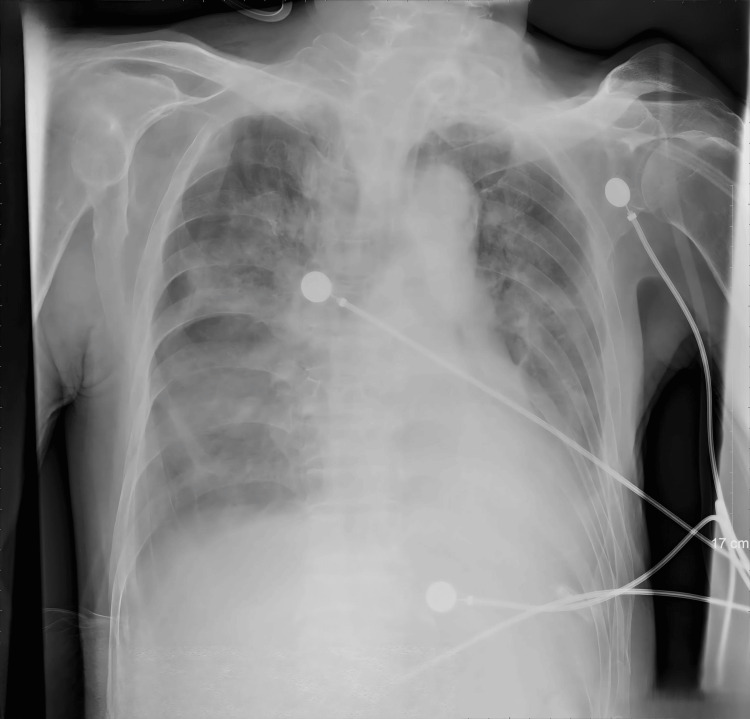
Bedside anteroposterior (AP) chest radiograph showing bilateral pneumonia with small bilateral pleural effusions

After confirming the patient's stable vital signs (SpO_2_ 95%, ～ 99%, HR 75 bpm), the doctor removed the tube. Ten hours later, a chest X-ray demonstrated bilateral increased bronchovascular markings, patchy ill-defined opacities in both lungs, blurred diaphragmatic contours, and blunted costophrenic angles, consistent with bilateral pulmonary infection accompanied by minimal bilateral pleural effusion (Figure 2).

The naso-intestinal tube was reinserted via the esophagus into the stomach under laryngoscopy guidance. Throughout the clinical course, despite radiologically confirmed intrathoracic malposition of the naso-intestinal tube, chest radiographs and physical examination revealed no evidence of pneumothorax, free pleural air, or bronchopleural fistula. The patient was mechanically ventilated with a symmetric thoracic contour, no unilateral chest fullness, tracheal deviation, or subcutaneous emphysema. Auscultation revealed bilateral diffuse dry and wet rales, without pleural friction rub.

Subsequently, six hours later, the patient’s temperature normalized, SpO_2_ reached 100%, and the condition remained stable, allowing transfer out of the ICU. One day later, the placement of the naso-intestinal tube was verified by aspirating a small volume of fluid with a pH >7, indicating that the tube tip was positioned in the intestine. Enteral nutrition was initiated at a slow rate. One day after ICU discharge, the patient developed a fever with clinical features suggestive of sepsis. Given persistent febrile illness despite broad-spectrum antibacterial therapy (meropenem and amikacin), empiric antifungal therapy was initiated. Following treatment, the patient’s vital signs and hemodynamic status stabilized, no active source of infection was identified, and he was discharged six days later. 

## Discussion

The insertion of a feeding tube can be regarded as the most physiological method to enable enteral nutrition in patients who are unable to receive oral feeding [7]. According to the guidelines, patients who cannot tolerate gastric feeding or are at high risk of aspiration should undergo postpyloric feeding [2,3]. A randomized clinical trial has demonstrated that the insertion of nasoenteric tubes (NETs) frequently presents a substantial challenge in the ICU [8]. Studies have demonstrated an inadvertent insertion of nasogastric tubes (NGTs)/NETs into the airway, with a prevalence rate of up to 3.2% [5], leading to pneumothorax and thereby increasing morbidity, mortality, and the duration of hospital stay. Several interrelated factors contribute to this complication, and this case highlights a unique literature gap regarding silent misplacement in postradiotherapy NPC patients.

Anatomical and neural damage from NPC

NPC is particularly prevalent in East and Southeast Asia. Radiation therapy is the preferred treatment for NPC, with dysphagia being a common side effect [9]. During radiation therapy, normal tissues within the radiation field inevitably sustain varying degrees of damage, such as muscle and soft tissue fibrosis, as well as reduced muscle activity. Furthermore, cranial nerve damage may occur, leading to early functional disorders such as hypoglossal nerve damage causing tongue atrophy and paralysis, glossopharyngeal nerve damage resulting in pharynx and larynx dysfunction, and vagus nerve damage leading to soft palate paralysis. These effects typically manifest within six months to several years postradiation therapy [6].

This elderly patient had a 12-year history of radiation therapy for NPC and developed dysphagia and aspiration. Laryngoscopy revealed a diminished pharyngeal reflex, incomplete glottic closure, and weakened protective cough reflex. These conditions directly facilitated the silent entry of the naso-intestinal tube into the respiratory tract.

The rate of blind placement-related intrathoracic misplacement is 2%[10], but such cases in long-term postradiotherapy NPC patients are reported to be extremely rare, highlighting the uniqueness and clinical importance of this case.

Insertion of feeding tubes and confirmation of their positions

In clinical practice, blind tube placement is widely adopted due to its convenience and ease of execution. The success rate of blind placement varies between 35% and 100%[11]. Because the exact position is uncertain during insertion, approximately leading to 1.6% of placements unintentionally enter the lung [12]. This case reveals three critical and preventable errors: blind insertion, overreliance on unconfirmed aspiration, and delayed radiography.

The auscultation method involves injecting air into the feeding tube and listening for "gurgling" sounds over the epigastric region to verify its placement in the stomach. However, this technique is one of the least preferred [12], as a "pseudoconfirmatory gurgle" may still occur if the tube is misplaced in the respiratory tract or chest, just as occurred in this case [13]. The guideline recommends the pH method as the first-line approach for confirming the initial placement of the feeding tube [12]. A pH value ≤5.5 is considered safe, as it effectively excludes the risk of respiratory misplacement [14]. Most guidelines agree that radiography is the most accurate method for confirming the tip position of a newly inserted tube, particularly in high-risk patients [15]. As recommended by the American Society for Parenteral and Enteral Nutrition (ASPEN) guidelines, radiography is the gold standard for verifying the tube course after blind insertion, and non-radiographic methods alone cannot serve as definitive confirmation [16]. Critically ill patients should undergo radiographic confirmation to ensure the correct placement prior to initiating feedings [17]. 

In this case, after inserting the tube to a depth of 55 cm, a small volume of fluid was aspirated and erroneously identified as gastric juice without pH measurement. Subsequent auscultation after air injection further reinforced the misjudgment that the tube was correctly positioned in the stomach. This nine-hour delay in imaging confirmation represents a serious preventable error. Moreover, this case showed a completely silent misplacement without any warning signs, which is rarely emphasized in previous literature but is especially life-threatening in NPC patients.

Diameter of the nasoenteric tube

There are reports indicating that narrow-bore feeding tubes may increase the risk of pneumothorax due to their small diameter [18]. Specifically, the lower the ratio of the feeding tube diameter to the tracheal diameter, the higher the likelihood of misplacement into the trachea, while the short-term effect on the patient's ventilation function becomes less noticeable. In this study, a nasoenteric feeding tube with an outer diameter of 3.23-3.38 mm (approximately one-eighth of the tracheal diameter) was used. When such a fine tube was mistakenly placed in the trachea, the patient exhibited high tolerance, a mild reaction, and unchanged oxygenation, which directly caused silent misplacement and delayed detection.

Presence of the tracheal cannula

When inserting a feeding tube, instruct the patient to swallow after inserting 10-15 cm. If needed, have them sip warm water while advancing the tube [4]. The presence of the tracheal cannula in these patients restricts laryngeal movement and impairs swallowing efficiency. Additionally, it reduces vocal cord closure capability. These factors collectively increase the risk of tube misplacement. Furthermore, the misplacement of the feeding tube into the trachea may result in hoarseness or an inability to speak. Additionally, the presence of an indwelling tracheal cannula precludes phonation due to the absence of airflow through the glottis. Following the inadvertent placement of the naso-intestinal tube into the trachea, the patient exhibited no typical symptoms such as cyanosis, choking cough, or a decrease in SpO_2_ levels. This explains why typical warning signs were absent, another important mechanism of silent misplacement unique to patients with tracheal cannulas.

Key learning points

Patients with NPC after radiotherapy carry a particularly high risk of silent feeding tube misplacement, which may occur without typical warning signs such as cough, cyanosis, or decreased oxygen saturation. Traditional bedside methods relying only on abdominal auscultation and simple gastric fluid aspiration without pH testing are unreliable and unsafe for confirming tube position. Although pH testing of aspirated contents with a threshold of pH ≤ 5.5 is a necessary initial verification step, it cannot serve as sufficient evidence alone to confirm correct placement. Blind bedside tube insertion should be avoided in patients with radiation-induced pharyngeal structural and functional impairment. Chest radiography should be arranged immediately after tube insertion, and enteral feeding should never be started until imaging has confirmed proper tube position.

## Conclusions

This case highlights that patients with NPC who have undergone radiotherapy and subsequently developed muscle and nerve damage are at increased risk of dysphagia and aspiration. Particular vigilance is required because thin tubes and tracheal cannulation greatly increase the risk of silent, unrecognized misplacement. To prevent catastrophic complications, pH testing must be performed immediately, and chest radiography (the gold standard) must be done right after insertion, not delayed, before any feeding. The core take-home message is that standardized, protocol-based insertion and immediate imaging confirmation are essential to prevent silent, life-threatening misplacement in high-risk NPC patients. 

## References

[1] Wischmeyer PE (2017). Tailoring nutrition therapy to illness and recovery. Crit Care.

[2] Singer P, Blaser AR, Berger MM (2023). ESPEN practical and partially revised guideline: clinical nutrition in the intensive care unit. Clin Nutr.

[3] Taylor BE, McClave SA, Martindale RG (2016). Guidelines for the provision and assessment of nutrition support therapy in the adult critically ill patient: Society of Critical Care Medicine (SCCM) and American Society for Parenteral and Enteral Nutrition (A.S.P.E.N.). Crit Care Med.

[4] Kaplan H, Curd D (2023). Safety of blind versus guided feeding tube placement: misplacement and pneumothorax risk. Intensive Crit Care Nurs.

[5] Motta AP, Rigobello MC, Silveira RC, Gimenes FR (2021). Nasogastric/nasoenteric tube-related adverse events: an integrative review. Rev Lat Am Enfermagem.

[6] Li JC, Mayr NA, Yuh WT, Wang JZ, Jiang GL (2006). Cranial nerve involvement in nasopharyngeal carcinoma: response to radiotherapy and its clinical impact. Ann Otol Rhinol Laryngol.

[7] Compher C, Bingham AL, McCall M, Patel J, Rice TW, Braunschweig C, McKeever L (2022). Guidelines for the provision of nutrition support therapy in the adult critically ill patient: the American Society for Parenteral and Enteral Nutrition. JPEN J Parenter Enteral Nutr.

[8] Illias AM, Hui YL, Lin CC, Chang CJ, Yu HP (2013). A comparison of nasogastric tube insertion techniques without using other instruments in anesthetized and intubated patients. Ann Saudi Med.

[9] Lan Y, Ohkubo M, Berretin-Felix G, Sia I, Carnaby-Mann GD, Crary MA (2012). Normalization of temporal aspects of swallowing physiology after the McNeill dysphagia therapy program. Ann Otol Rhinol Laryngol.

[10] Matsuda R, Kou Y, Kogita Y, Sakamaki Y (2025). Fistulous empyema due to bronchopulmonary laceration with a misintubated nasogastric tube: a case report. Gen Thorac Cardiovasc Surg Cases.

[11] Wang Q, Xuan Y, Liu C, Lu M, Liu Z, Chang P (2021). Blind placement of postpyloric feeding tubes at the bedside in intensive care. Crit Care.

[12] Metheny NA, Krieger MM, Healey F, Meert KL (2019). A review of guidelines to distinguish between gastric and pulmonary placement of nasogastric tubes. Heart Lung.

[13] Phukpattanachai K, Praditseree N, Skjolaas S, Klaychaiya S, Trongtrakul K (2024). Accuracy of pH strip testing and pH liquid testing versus standard pH meter of gastric contents in critically ill patients: a diagnostic accuracy study. BMJ Open.

[14] Ni MZ, Huddy JR, Priest OH, Olsen S, Phillips LD, Bossuyt PM, Hanna GB (2017). Selecting pH cut-offs for the safe verification of nasogastric feeding tube placement: a decision analytical modelling approach. BMJ Open.

[15] Macedo AB, Assis MC, Milioni KC, Canto DF, Souza CM, Chaves EH (2021). Elaboration and validation of a protocol for safe administration of enteral nutrition in hospitalized patients. Rev Gaucha Enferm.

[16] Boullata JI, Carrera AL, Harvey L (2017). ASPEN safe practices for enteral nutrition therapy. JPEN J Parenter Enteral Nutr.

[17] Powers J, Brown B, Lyman B (2021). Development of a competency model for placement and verification of nasogastric and nasoenteric feeding tubes for adult hospitalized patients. Nutr Clin Pract.

[18] Freeberg SY, Carrigan TP, Culver DA, Guzman JA (2010). Case series: tension pneumothorax complicating narrow-bore enteral feeding tube placement. J Intensive Care Med.

